# Novel Inhibitors of SARS-CoV-2 RNA Identified through Virtual Screening

**DOI:** 10.1021/acs.jcim.4c00758

**Published:** 2024-07-22

**Authors:** Gregory Mathez, Andrea Brancale, Valeria Cagno

**Affiliations:** † Institute of Microbiology, University Hospital of Lausanne, University of Lausanne, 1011 Lausanne, Switzerland; ‡ Department of Organic Chemistry, University of Chemistry and Technology Prague, 16628 Prague 6, Czech Republic

## Abstract

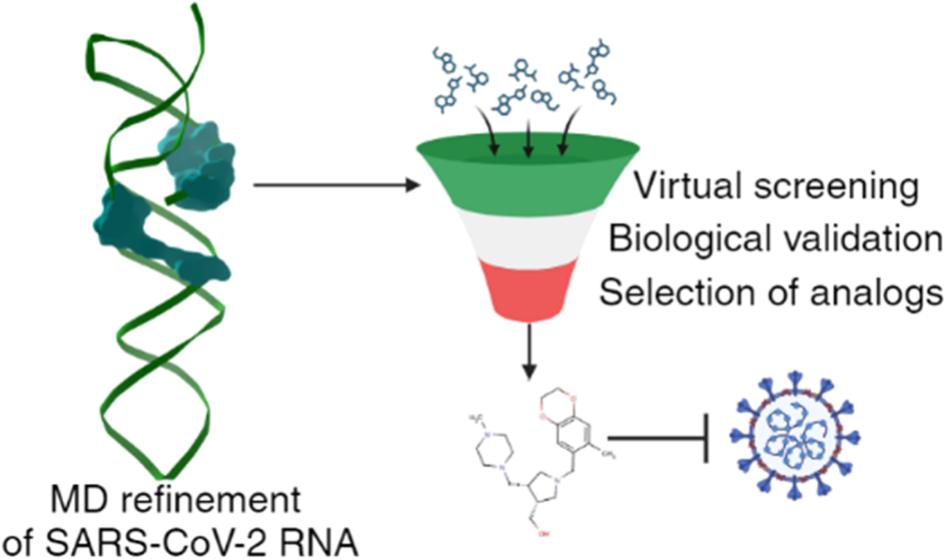

We currently lack antivirals for most human viruses. In a quest for new molecules, focusing on viral RNA, instead of viral proteins, can represent a promising strategy. In this study, new inhibitors were identified starting from a published crystal structure of the tertiary SARS-CoV-2 RNA involved in the −1 programmed ribosomal frameshift. The pseudoknot structure was refined, and a virtual screening was performed using the repository of binders to the nucleic acid library, taking into consideration RNA flexibility. Hit compounds were validated against the wild-type virus and with a dual-luciferase assay measuring the frameshift efficiency. Several active molecules were identified. Our study reveals new inhibitors of SARS-CoV-2 but also highlights the feasibility of targeting RNA starting from virtual screening, a strategy that could be broadly applied to drug development.

## Introduction

For most known viruses, antivirals are still lacking, leading to morbidity and mortality, particularly among the immunocompromised and the elderly. Current methods target either viral or host proteins. Unfortunately, some proteins are undruggable, and inhibiting host proteins can lead to toxicity. Nowadays, a promising strategy is to target viral RNA (reviewed by Mathez and Cagno^1^). Tertiary RNA structures conserved among viral families, and distinct from those in human RNA, can be targeted, resulting in small molecules with broad activity and reduced off-targets.^2^

In late 2019, the discovery of severe acute respiratory syndrome coronavirus 2 (SARS-CoV-2) initiated efforts to develop vaccines and treatments aimed at managing the pandemic and reducing associated mortality. An interesting RNA target of SARS-CoV-2 is its −1 programmed ribosomal frameshift (−1PRF) element.^3,4^ The virus employs −1PRF to regulate the expression of viral proteins crucial for its replication such as the RNA-dependent RNA polymerase. SARS-CoV-2 has two overlapping open reading frames (ORFs) in its genome, denoted as ORF1a and ORF1b. The ORF1a ends with a stop codon. However, through −1PRF, the stop codon can be bypassed, allowing for the translation of the entire ORF1ab and enabling the synthesis of the replication machinery encoded within ORF1b.^1,3,5^ It is estimated that a −1PRF occurs in 45–69% of the translation events.^6^ The elements required in vitro for −1PRF include a specific slippery sequence on the viral RNA in conjunction with the formation of a tertiary RNA structure. The conformation of this RNA, determined in close proximity to ribosome during translation, was reported to be an H-type pseudoknot structure.^3,7−10^ Since this arrangement is uncommon in humans, the risk of off-target effects is reduced, and targeting this RNA structure holds a significant interest.^1^

Several small molecules affecting −1PRF of SARS-CoV-2 have been previously identified, demonstrating the feasibility of the approach. Specifically, merafloxacin,^3,11,12^ a fluoroquinolone, and geneticin,^12^ an aminoglycoside, showed specific inhibition of the −1PRF of SARS-CoV-2. Geneticin, in our work, was shown to inhibit various variants of concern, and its effectiveness was maintained in human-derived respiratory tissues. Furthermore, through molecular docking, the putative binding site of geneticin in the frameshifting element was identified and validated via mutagenesis. Notably, a combination of three mutations in the pseudoknot structure, aimed to occlude the binding site, led to decreased inhibitory activity of the frameshift mediated by both merafloxacin and geneticin.

However, fluoroquinolones and aminoglycosides are antibiotics associated with toxicity in humans,^13−17^ thus hindering their development as antivirals. To understand the requirements for −1PRF inhibitors devoid of toxicity, new compounds targeting this mechanism must be identified.

In this study, a new crystal structure of the tertiary viral RNA structure involved in the −1PRF of SARS-CoV-2^7^ was refined by molecular dynamics. Based on the binding site identified by our previous study on geneticin’s activity, a virtual screening on different structures was performed using an experimentally verified library of compounds targeting RNA. Selected molecules were used against an omicron variant of SARS-CoV-2 and in a dual-luciferase assay to confirm the inhibition of −1PRF element of the virus leading to the discovery of novel SARS-CoV-2 inhibitors.

## Materials and Methods

### Cells and Viruses

Vero E6 cells (ATCC CRL-1586) were provided by Prof. Gary Kobinger and maintained in Dulbecco’s modified Eagle’s medium (DMEM) with GlutaMAX, high glucose supplemented (Gibco) with 10% fetal bovine serum (FBS) (Pan Biotech), and penicillin/streptavidin (Gibco).

Omicron (BA.1) SARS-CoV-2 virus was isolated from a clinical sample from the University Hospital of Lausanne using the method of Mathez and Cagno.^18^

### Compounds

Compounds used were purchased from ChemBridge except for piperaquine (Sigma-Aldrich), merafloxacin (MedChemExpress), and geneticin (Promega). Geneticin was dissolved in water, and all other molecules were dissolved in dimethyl sulfoxide (DMSO) (Sigma-Aldrich). The first three digits of the commercial ID of ChemBridge or the commercial name are used in the text and the figures.

### Refinement of Pseudoknot

Crystal structures of the pseudoknot (PDB 7LYJ and 7MKY(7)) were chosen. 7MKY was repaired due to a missing nucleobase utilizing 7LYJ as a template. Both structures were then refined through molecular dynamics using GROMACS 2023.1.^19−24^ Topology was generated using Amber ff99bsc0χOL3 force field,^25−30^ and tip3p water was used. A cubic box was filled with NaCl with a concentration of 15 mM and a neutral charge. Energy minimization was carried out for 50,000 steps, with the temperature and pressure equilibrium 100 and 10 ps, respectively. Molecular dynamics were then conducted for 500 ns at 300 K and 1 bar. Positions were recorded at every 100 ps. The trajectory was then centered. Clustering was performed by using the GROMOS method with a cutoff of 0.15 nm. Trajectory and clustered structures were visualized using VMD version 1.94a55.^31^

### Virtual Screening

Virtual screening was conducted on Maestro Schrodinger version 2023-2.^32^ The repository of binders to nucleic acids (ROBIN) library^33^ was prepared using LigPrep by generating possible states at pH 7.0 ± 2 with Epik 7 and with OPLS4 force field. Tautomeric forms and chiral centers specified in the input structures were preserved, while for newly generated chiral centers (e.g., quaternary nitrogen atoms on asymmetric pyrrolidine), all possible configurations were included. Overall, LigPrep enumeration resulted in 3932 structures out of the 2003 input structures. Four clusters were chosen as the input structures. The grids were generated on each cluster using a box of 20 and 30 Å as inner and outer boxes, respectively, centered on the centroid of specified residues (G3, U4, G5, U6, U29, A57, and U58). The virtual screening workflow was then independently applied to each grid in the prepared library. After the high-throughput virtual screening, 25% of the best drugs were kept for the subsequent docking steps. After SP and XP docking, 50% of the best compounds were retained. The final poses were then refined by Prime MMGBSA. Compounds binding at least three structures were visually inspected for the final selection.

### Antiviral Activity

The antiviral activity of selected compounds was evaluated with a protocol similar to the one described previously.^12,18^ Vero E6 cells (10^5^ cells in a 24-well plate) were infected with 100 plaque-forming units of SARS-CoV-2. After 1 h of incubation, the inoculum was removed, and an overlay of 0.6% Avicel gp3515 (Selectchemie AG) and 2.5% FBS in DMEM containing serial dilutions of the drug was added. Three days later, cells were fixed with 4% formaldehyde (Sigma-Aldrich) followed by staining with crystal violet (Sigma-Aldrich). Plaques were manually counted.

### Dual Luciferase

The frameshift efficiency was assessed using a dual-luciferase assay as described previously.^3,12^ Vero E6 cells (10^4^ cells in a 96-well plate) were treated with a single nontoxic dose of the compound in 10% FBS DMEM. Wild-type or in-frame control plasmids were transfected using Lipofectamine 3000 (Invitrogen). The following day, cells were washed with phosphate-buffered saline (PBS) and passively lysed. The activity of firefly and renilla luciferase was assessed with a Dual-Luciferase Reporter Assay System kit (Promega) in a white plate using a luminometer (TriStar LB 941, Berthold Technologies). Frameshift efficiency was calculated as done by Bhatt et al.^3^

### Cell Viability

Following the protocol of Varricchio and Mathez et al.,^12^ compounds were added to Vero E6 cells in serial dilution in 2.5% FBS DMEM. Three days later, cells were washed with DMEM and a final concentration of 0.5 mg/mL of 3-(4,5-dimethyl-2-thiazolyl)-2,5-diphenyl-2*H*-tetrazolium bromide (MTT) (Sigma-Aldrich) in DMEM was added for 3 h at 37 °C. Cells were lysed with DMSO, and absorbance was read at 570 nm (BioTek Epoch 2, Agilent).

### Molecular Dynamics with Ligand

Molecular dynamics of the SARS-CoV-2 pseudoknot structure with ligand was conducted following the procedure mentioned above for the refinement of the structure. Ligand topology was generated using the ACPYPE server^34,35^ and was added to the topology of SARS-CoV-2 RNA. RNA and the ligand were restrained simultaneously for the temperature and pressure equilibrium.

### Docking of 194 Analogues

Compound **782** was prepared following the same procedure as that for the preparation of the ROBIN, as described above. The analogue was docked using compound **194** as the center of the grid on cluster A. An XP docking protocol was used followed by Prime MMGBSA refinement. Results were visualized and analyzed in MOE 2022.02.^36^

### Statistics

Biological experiments were performed in duplicate or quadruplicate for the dual-luciferase assay and at least two independent experiments. Results are shown as mean and standard error of the mean (SEM). The effective concentration inhibiting 50% (EC_50_) and the concentration reducing cell viability by 50% (CC_50_) values were calculated by regression analysis using the program GraphPad Prism version 10.1.2 to fit a variable slope sigmoidal dose–response curve as described by Mathez and Cagno.^18^ One-way analysis of variance (ANOVA) followed by multiple comparison analysis was used as a statistical test to compare grouped analysis.

## Results

### Refinement and Clustering of SARS-CoV-2 Pseudoknot

**Figure 1:**
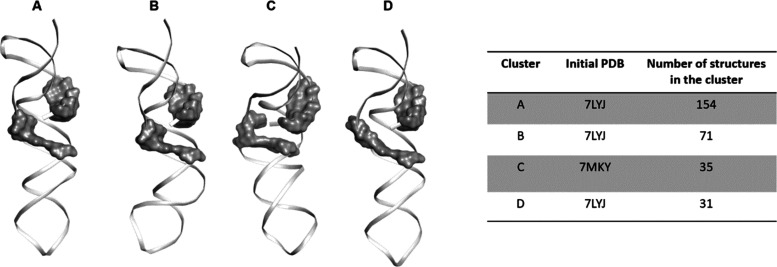
Selected cluster for virtual screening. Four representative clusters, derived from clustering using a cutoff of 0.15 nm of the 500 ns molecular dynamics of PDB 7LYJ and 7MKY, were selected for the virtual screening. The surface area represents specified residues previously found to be in geneticin’s pocket. The same residues were used to define the grid for the virtual screening.

Recently, a high-resolution model of the pseudoknot of SARS-CoV-2 was determined (PDB: 7LYJ (2.11 Å) and 7MKY (1.31 Å)).^7^ To consider RNA flexibility in the virtual screening, this structure underwent refinement through a 500 ns molecular dynamics simulation using GROMACS (Figure S1). Following the simulation, the various frames were clustered. Four representative clusters were selected to take into account RNA flexibility at the binding site (Figure 1).

### Selection of Small Molecules Binding SARS-CoV-2 Pseudoknot

Potential sites on the pseudoknot of SARS-CoV-2 were identified using Site Finder of MOE. Two sites were in close proximity and involved nucleotides previously discovered in the binding site identified in our prior study with geneticin.^12^ These two potential sites were merged into a single binding site for a virtual screening. In the ROBIN library,^33^ compounds previously shown to bind RNA experimentally and not DNA, and having drug-like properties, were docked against each selected cluster. Molecules binding to at least 3 clusters were visually inspected (21 molecules). One molecule was excluded due to its instability in water. Based on their interactions on the predicted pose and their chemical diversity, six commercially available molecules were purchased.

### Antiviral Evaluation of Selected Compounds against SARS-CoV-2

The selected compounds were evaluated for toxicity on Vero E6 cells measuring their viability after 3 days of exposure (Table 1, Figure S2). Piperaquine exhibited high toxicity with a CC_50_ of 5.66 μM. Compounds **188** and **229** (compound numbering refers to the first three digits of the compound ID of the ChemBridge library) were found to have a CC_50_ of 80.6 and 93.03 μM, respectively. Antiviral activities of the selected compounds were subsequently evaluated against an Omicron variant of SARS-CoV-2 previously isolated from a clinical sample (Table 1).

**Table 1:**
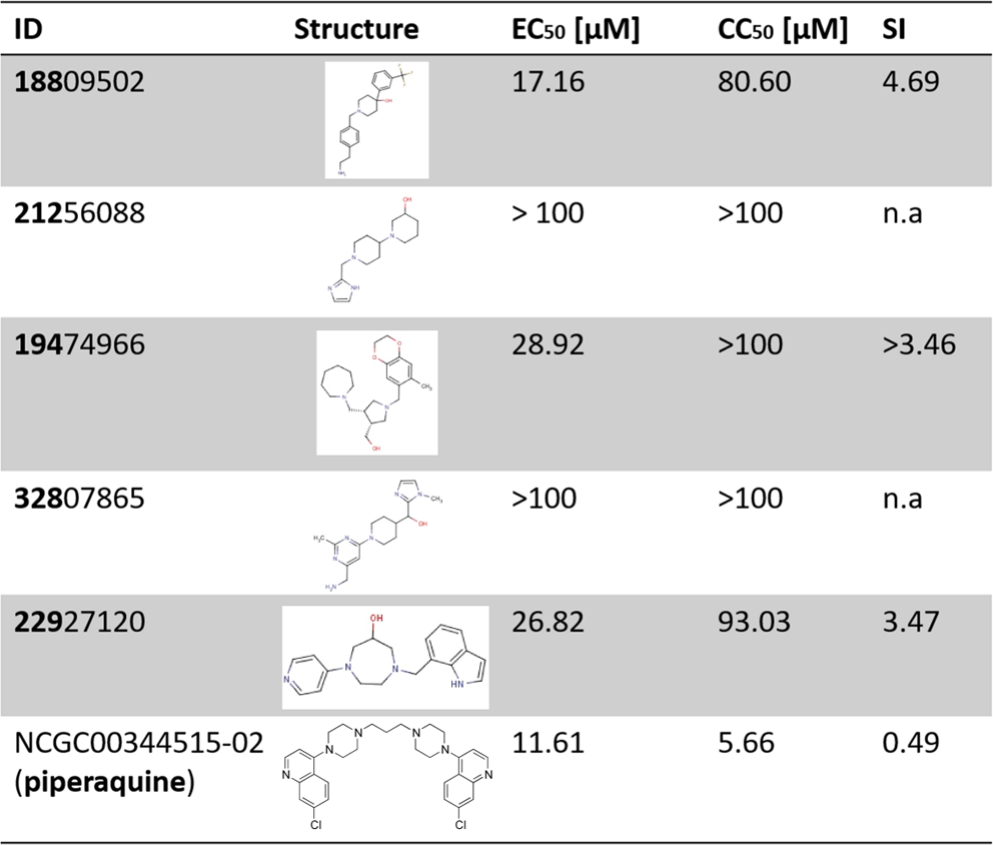
Antiviral Activity against SARS-CoV-2 and Cytotoxicity of Selected Molecules after Virtual Screening^a^

a Identification number used in ROBIN library (in bold the name used in the text); EC_50_, effective concentration inhibiting 50% of the virus; CC_50_, concentration reducing cell viability by 50%; SI, selective index.

**Figure 2:**
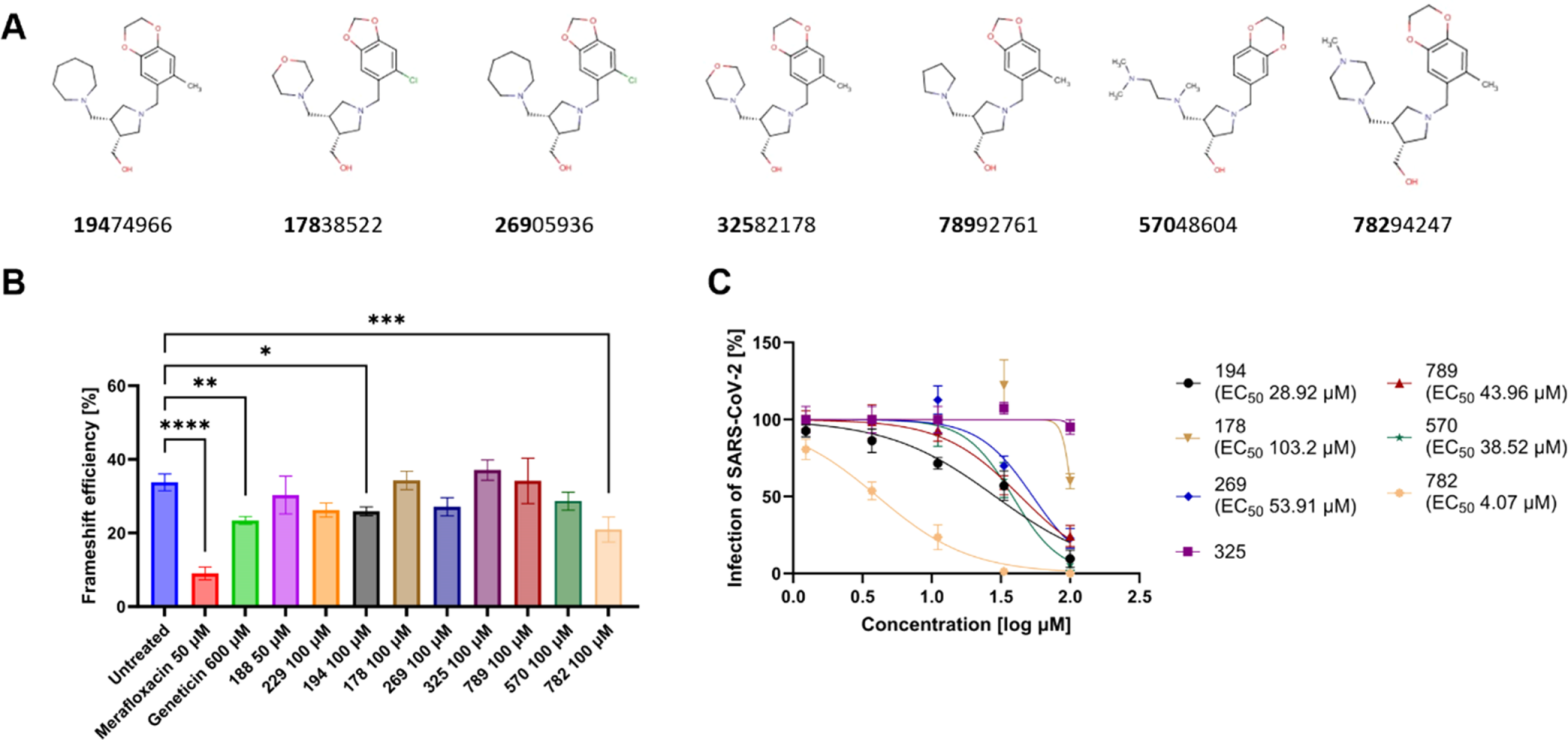
Frameshift efficiency and antiviral activity of compound **194** analogues. (A) Analogues of **194** are represented. The first three digits of the ID number of the molecule (in bold) are used in the text and figures. (B) Dual-luciferase assay was conducted on Vero E6 cells. Frameshift efficiency was evaluated in the presence of inhibitors identified after the virtual screening and analogues of compound **194**. Merafloxacin and geneticin were used as positive controls. (C) Antiviral activity of analogues of compound **194** was evaluated against an Omicron variant of SARS-CoV-2 on Vero E6 cells. EC_50_: effective concentration inhibiting 50% of the virus. *P < 0.0332, **P < 0.0021, ***P < 0.0002, ****P < 0.0001.

Compounds **188**, **194**, and **229** showed EC_50_ values of 17.16, 28.92, and 26.82 μM, respectively. To verify the specificity of the activity on the −1PRF of SARS-CoV-2, a dual-luciferase assay was performed. In this assay, the minimal frameshifting element of SARS-CoV-2 is cloned between two luciferases. The upstream luciferase is constitutively expressed while the downstream one is expressed only upon frameshifting. As a control, an in-frame construct was used where both luciferases are expressed. Additionally, two positive controls, merafloxacin and geneticin previously shown to reduce frameshifting, were included in the assay. With this method, only compound **194** showed a significant reduction in frameshift efficiency, while compound **229** showed a similar reduction but was not significant (Figure 2A,B).

### Analysis and Optimization of Compound **194**

**Figure 3:**
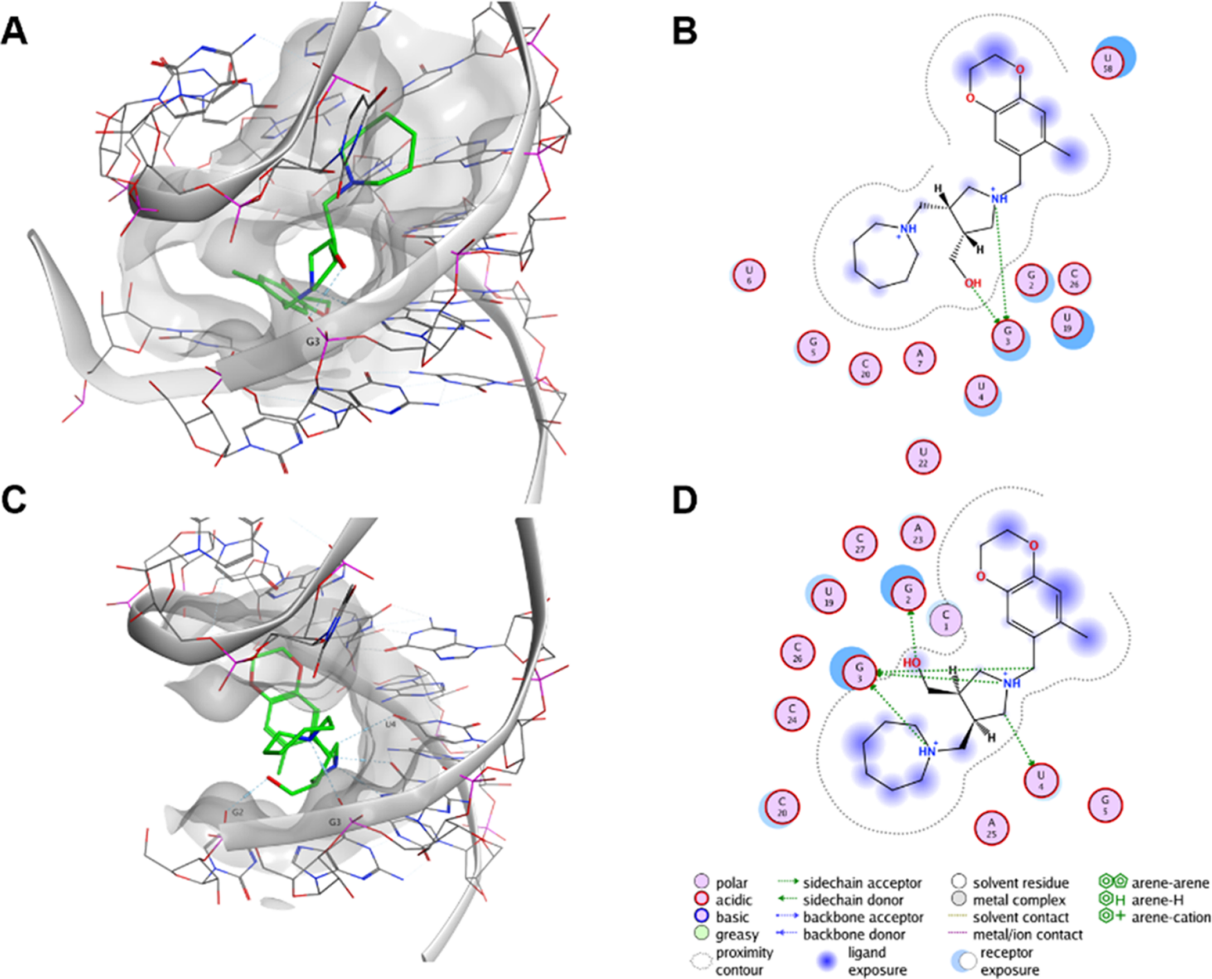
Binding pose of compound **194**. (A, B) Binding pose of **194** on cluster A after the virtual screening. (C, D) Binding pose of **194** on cluster A after molecular dynamics of 500 ns represented by the most populated clusters after clustering with a cutoff of 0.15 nm. (A, C) Site view and (B, D) ligand interaction maps were done with MOE.

Compound **194** from our first set showed the most potent antiviral activity, displaying a reduction of frameshift efficiency and lower cytotoxicity. Consequently, it underwent further analysis. Starting from the initial binding pose postvirtual screening, a 500 ns molecular dynamic of the pseudoknot and compound **194** was performed (Figure S3). The ligand maintained its position and appeared to interact with several nucleotides such as G2, G3, and U4 through hydrogen bonds (Figure 3). G3 was previously identified as a main interactor from the binding pose postvirtual screening.

Commercially available analogues of compound **194** were purchased. Their cytotoxicity, antiviral activity, and frameshift efficiency were assessed (Figure 2). While the CC_50_ for all compounds exceeded 100 μM (Figure S2), one of the analogues, compound **782**, showed a more potent antiviral activity (EC_50_ 4.07 μM) and a significant and improved reduction of frameshift efficiency compared to **194**; furthermore, the inhibition was shown to be dose-dependent (Figure S4). Compound **782** was docked on cluster A by using compound **194** as the center of the grid. The additional protonated nitrogen showed interaction with the SARS-CoV-2 pseudoknot (Figure S5). Other analogues showed inhibitory activity against SARS-CoV-2 but without reducing frameshift except compound **269**, which showed a nonsignificant reduction of frameshift efficiency. Compound **325** in contrast did not inhibit wild-type virus.

## Discussion

SARS-CoV-2 relies on a programmed ribosomal frameshift to regulate the expression of viral proteins crucial for replication. By disrupting this key process, small molecules can impede the life cycle of the virus. To this end, the antiviral activity of molecules acting on the frameshift stimulating element responsible for the −1PRF of SARS-CoV-2 was evaluated.

During infection, in proximity of the ribosome translating the end of ORF1a, a pseudoknot structure is believed to be involved in the −1PRF mechanism.^3,7,10^ This structure was recently crystallized with high resolution and therefore represents the most accurate model obtained.^7^ This structure was refined by molecular dynamics, and the resulting trajectory was clustered (Figures 1 and S1). A virtual screening was performed on rigid RNA structures with RNA flexibility considered by docking the library of molecules independently on different structures. This screening was validated by assessing the antiviral activity against the Omicron variant of SARS-CoV-2 and the inhibitory effect on the frameshift efficiency with a dual-luciferase assay (Table 1, Figure 2). Piperaquine, an antimalarial drug previously shown to be active against SARS-CoV-2,^37^ was among the molecules identified. However, due to its high toxicity (Figure S2) and a small selective index (Table 1), piperaquine was not further retained for analysis. Nonetheless, more promising inhibitors were discovered. Notably, compound **194** showed an EC_50_ of 28.92 μM and significantly reduced the frameshift efficiency by 23% (Table 1, Figure 2). Importantly, this molecule bound to all clusters in the initial virtual screening. Following molecular dynamics simulation, the interaction of **194** with the frameshifting element was confirmed (Figures 3 and S3).

To explore the potential enhancement of the original molecule’s activity and understand the functional groups required for inhibition, analogues of **194** were evaluated. Compound **782** showed an EC_50_ of 4.07 μM and significantly reduced the frameshift efficiency by 38% (Figures 2 and S4). Notably, the inhibition of this compound on the wild-type virus is more potent than previously reported inhibitors, such as merafloxacin and geneticin.^12^

Although the complete structural relationship with antiviral activity is not yet understood, intriguing observations were observed following minor chemical modifications of compound **194**, suggesting potential selectivity against the SARS-CoV-2 pseudoknot (Figure 2). Compound **325**, substituting an azacycloheptane with a morpholine, exhibited a loss of antiviral activity against SARS-CoV-2. This loss was corroborated by compound **178**, which despite harboring an additional modification with a 5-chloro-1,3-benzodioxole instead of a 2,3-dihydro-6-methyl-1,4-benzodioxin, also experienced a decrease in potency. Compound **269**, undergoing the same modification, retained activity against the wild-type virus albeit with reduced potency and remained active in the dual-luciferase assay with a reduction in frameshift, though not statistically significant. Conversely, replacing the azacycloheptane in compound **782** with a methylpiperazine improved potency and had a favorable impact on the frameshift reduction. Protonation of the added nitrogen might facilitate additional interactions with the RNA backbone, as evidenced by docking the compound onto SARS-CoV-2 cluster A (Figure S5), thereby potentially explaining the enhanced antiviral activity. This is also supported by the results obtained when docking morpholine analogue **325**. This compound, likely due to the presence of the oxygen atom instead of the basic NMe group, might not be able to interact efficiently with the RNA phosphate groups. This is reflected in the docking results, where **325** shows a much lower score than that of **782**. It should be noted that all possible ionization states at pH 7 ± 2 were generated for each structure, including asymmetric protonation on the pyrrolidine ring, using Epik 7 (Schrödinger suite). Subsequently, all of the generated structures were docked into the RNA. The results shown in Figure S5 represent the best-scored protonated combination of **782**. Although this fully protonated form might not represent the most abundant species in solution (calculated p*K*_a_ values for **782** are shown in Figure S6), it is possible that this state is present in a productive concentration when binding to the RNA, as observed, for example, with polyamines binding to nucleic acids.^38^

It is important to acknowledge the limitations of our study linked to the use of the −1PRF crystal structure as a starting point for the virtual screening. The pseudoknot structure was identified several times by structural analysis^7−9^ even in complex with a ribosome^3^ or by simulation showing that this structure is the only viable conformation when frameshift occurs.^10^ Therefore, it is a valuable target. However, this region of the SARS-CoV-2 RNA is reported to have multiple possible conformations. By assessing the frameshifting element structure in infected cells,^39−41^ the pseudoknot structure was not observed, possibly due to long-range interactions with other viral RNA segments. Therefore, during infection, small molecules targeting the frameshifting element might encounter other structures for which they might have a low affinity. Applying virtual screening on the different models could expand the inhibitors of SARS-CoV-2 by altering −1PRF by different mechanisms.

Another limitation is linked to the absence of RNA modifications in the starting model. SARS-CoV-2 RNA can undergo modifications, such as *N*^6^-methyladenosine, 5-methyl cytosine, pseudouridine, or 2′-*O*-methylation.^40,42−45^ These modifications could potentially alter the structure or alter the binding interactions.

Furthermore, in our study, the initial compounds were selected to bind to at least three clusters. Previous studies, aimed at identifying antivirals targeting RNA, selected molecules binding single clusters from a larger set of structures.^46,47^ By performing a similar protocol, the initial set of molecules could be expanded by selecting additional potential inhibitors.

Our research has unveiled novel inhibitors of SARS-CoV-2 altering the −1PRF mechanism of the virus, giving insights into the requirements for inhibiting SARS-CoV-2 RNA with drug-like compounds. Importantly, to the best of our knowledge, our study identified the most potent −1PRF SARS-CoV-2 inhibitor to date and laid the groundwork for a broader approach aimed at targeting viral RNA using small molecules.

## Data Availability

The data underlying this study are openly available in Zenodo at 10.5281/zenodo.11098463.

## Supporting Information Available

Trajectory of crystal structures of SARS-CoV-2 pseudoknot; toxicity of selected compounds and analogues on Vero E6 cells; trajectory of SARS-CoV-2 cluster A with compound **194**; frameshift efficiency of compound **782**; binding pose of compound **782**; calculated p*K*_a_ for compound **782** (PDF)

## Abbreviations

SARS-CoV-2: severe acute respiratory syndrome coronavirus 2
ROBIN: repository of binders to nucleic acids
ORF: open reading frame
DMEM: Dulbecco’s modified Eagle’s medium
FBS: fetal bovine serum
MTT: 3-(4,5-dimethyl-2-thiazolyl)-2,5-diphenyl-2*H*-tetrazolium bromide
CC_50_: concentration reducing cell viability by 50%
EC_50_: effective concentration inhibiting 50% of the virus
SI: selective index
RMSD: root mean square deviation
