# Supplementary material for: Novel Inhibitors of SARS-CoV-2 RNA Identified through Virtual Screening
Source: J Chem Inf Model. 2024 Jul 22;64(15):6190–6. doi: 10.1021/acs.jcim.4c00758 (PMC11323243; doi:10.1021/acs.jcim.4c00758)
Supplement: Supplementary file 1 — ci4c00758_si_001.pdf [file ci4c00758_si_001.pdf]

SUPPLEMENTARY INFORMATION

Novel Inhibitors of SARS-CoV-2 RNA Identified through Virtual Screening

Gregory Mathez[1,2], Andrea Brancale[2*#], Valeria Cagno[1*#]

*co-last author

#corresponding authors (andrea.brancale@vscht.cz; valeria.cagno@chuv.ch)

1 Institute of Microbiology, University Hospital of Lausanne, University of Lausanne, 1011 Lausanne, Switzerland

2 Department of Organic Chemistry, University of Chemistry and Technology Prague, 16628 Prague 6, Czech Republic

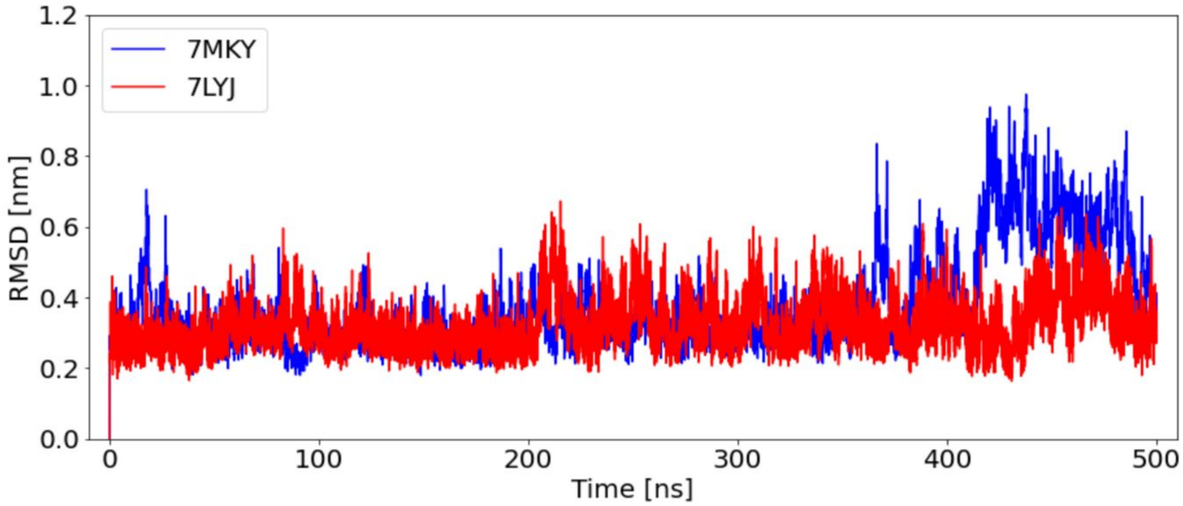

Figure S1 Trajectory of crystal structures of SARS-CoV-2 pseudoknot. PDB 7MKY and 7LYJ were used as the initial structure for molecular dynamics of 500 ns using GROMACS. Root mean square deviation (RMSD) was calculated for each frame relative to the crystal structure and represented in the graph according to the time of the simulation.

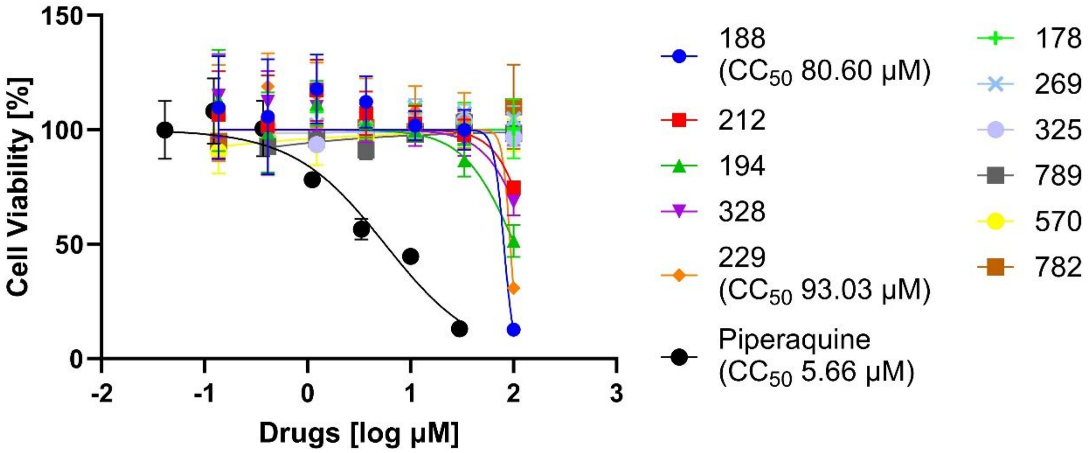

Figure S2 Toxicity of selected compounds and analogs on Vero E6 cells. Cells were treated with the different molecules from 100 μM for 3 days at 37˚C. Cell viability was measured by the absorbance of MTT. CC_{50} was calculated with GraphPad Prism.

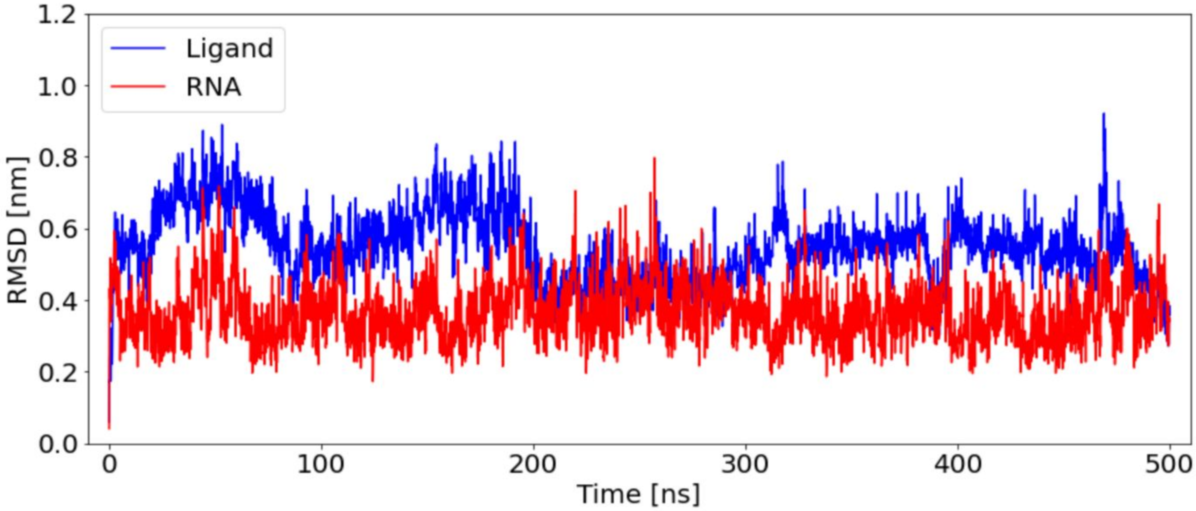

Figure S3 Trajectory of SARS-CoV-2 cluster A with compound 194. Compound **194** docked in SARS-CoV-2 cluster A after virtual screening was used as initial structure molecular dynamics of 500 ns using GROMACS. RMSD of the ligand and RNA were calculated for each frame relative to the input structure and represented in the graph according to the time of the simulation.

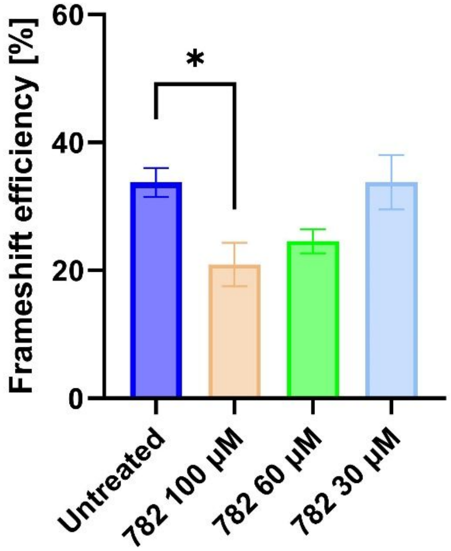

Figure S4 Frameshift efficiency of compound 782. Dual luciferase assay was conducted on Vero E6 cells. Frameshift efficiency was evaluated in the presence of different concentrations of compound 782. * $P < 0.0332$

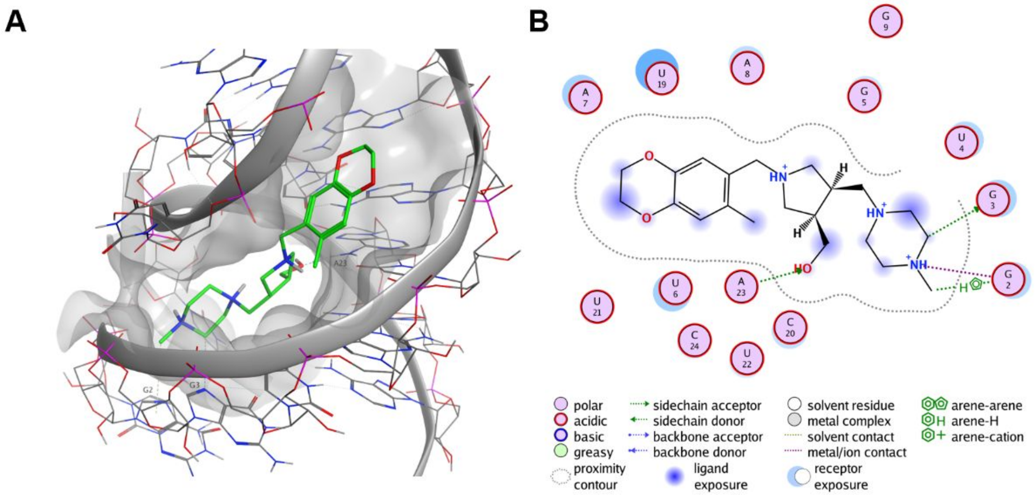

Figure S5 Binding pose of compound 782. A) Site view of the binding pose of **782** on cluster A docked on the binding site of compound **194**. B) Ligand interactions map was done with MOE.

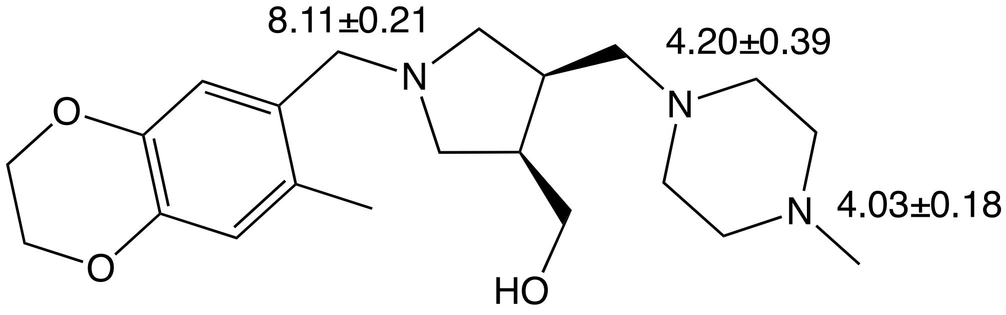

Figure S6 Calculated pKa for compound 782.
